# Adjuvantation of Kyasanur Forest Disease vaccine with TLR9 agonist CpG adjuvant enhances immunological efficacy and potency of the vaccine

**DOI:** 10.1371/journal.pone.0329348

**Published:** 2025-07-31

**Authors:** Chandranaik B. Marinaik, Bharath T. Lakshmikanth, Chinmayie K. Siddaramegowda, Apsana Rizwan, Amitha Reena Gomes, Keerthi Kaje, Doddamane Rathnamma, Shrikrishna Isloor, Archana M, Jagadish Hiremath, Raveendra Hegde

**Affiliations:** 1 Institute of Animal Health and Veterinary Biologicals, KVAFSU, Bangalore, India; 2 Veterinary College, KVAFSU, Bangalore, Karnataka, India; 3 ICAR-National Institute of Veterinary Epidemiology and Disease Informatics, Bangalore, India; Banaras Hindu University Institute of Medical Sciences, INDIA

## Abstract

The present study was carried out with the objectives of formulation of Kyasanur Forest Disease (KFD) vaccine adjuvanted with TLR9 agonist, CpG; evaluation of cell mediated and humoral immune response of CpG adjuvanted KFD vaccine in mice. The KFD seed virus (P9605) procured from ICMR-National Institute of Virology, Pune was used for the preparation of KFD vaccine and was confirmed by Real Time Reverse Transcription-PCR. The KFD vaccine prepared in chick embryo fibroblast primary culture was inactivated with 0.1% of formalin. Safety test was performed to assess the safe does of CpG adjuvant in mice. The KFD vaccine adjuvanted with 10 µg of CpG per dose of vaccine was formulated and immunologically evaluated in mice. Evaluation of humoral immune response (HIR) by mouse protection test showed that CpG when used as an adjuvant, significantly (P < 0.0001) enhances HIR in mice with better survival percentages when compared to the non-adjuvanted KFD vaccine. The cell mediated immune response/cytokine response evaluated by quantifying Interferon-ϒ (IFN-ϒ), Tumour Necrosis Factor-Alpha (TNF-α) and IL-12 production using ELISA showed that CpG significantly (P < 0.001) increases IFN-ϒ, TNF- α and IL-12 when used as an adjuvant in KFD vaccine in mice. The potency test showed that CpG adjuvanted vaccine has higher protective efficacy with better protective index compared with the non-adjuvanted KFD vaccine. Taken together these findings, the study recommends CpG as an adjuvant in the formalin inactivated KFD vaccine since the adjuvantation was safe and enhanced KFD specific immunogenicity and protective efficacy in mice.

## Introduction

Vector borne diseases pose considerable public health problems worldwide. Many vector-borne diseases are emerging and re-emerging at increasing rate and are appearing in new regions in the past two decades [1,2]. Kyasanur Forest Disease (KFD) is one of the re-emerging vector borne diseases caused by Kyasanur Forest Disease Virus (KFDV) belonging to the virus family *Flaviviridae.* The KFDV is an enveloped, single-stranded, positive-sense RNA virus of about 45 nm diameter [3,4]. The KFD was first identified in Kyasanur forest in Soraba taluk of Shimoga district, India in 1957, where unusual deaths of red-faced bonnet macaques and black-faced langurs were reported with concurrent outbreaks in humans. The disease is mainly transmitted by the bite of infected hard tick *Haemaphysalis spinigera* [1,4].

After the discovery of KFD, various efforts were made to control the disease in India and several studies were performed to develop a potent vaccine against KFD. The KFDV was adapted to grow in chick embryo fibroblast cell culture and was used for KFD vaccine production [5]. A formalinized tissue culture vaccine was developed and employed as a vaccine against KFD in India [6] and is being used for control of the disease from past 50 years [1]. The vaccination schedule includes administration of two doses to 7–65 year age group in one month apart followed by a booster dose about 6–9 months after the primary vaccination thereafter, administration of annual booster dose is recommended for five years after the last confirmed case in the area [6,7]. The formalin inactivated tissue culture KFD vaccine is the only available vaccine in India produced by Institute of Animal Health and Veterinary Biologicals (IAH&VB), Bengaluru. This vaccine has greatly helped in reducing the morbidity and mortality due to KFD in India in past two decades [1]. The most recent epidemiological study [7] has found that the effectiveness of the currently available formalin inactivated non adjuvanted KFD vaccine is 62.4% among those who received one dose and 82.9% in those who received two doses of vaccine. Even though the currently available vaccine is effective and has helped in reducing the severity of infection; multiple doses of vaccine administration is causing the vaccine refusal in rural areas [7,8].

Adjuvants are the substances that non-specifically enhance the immune response when administered along with the antigens, they are added to vaccine to increase the immunogenicity of the vaccine [9]. The CpG ODNs are synthetic oligodeoxynucleotides (ODNs) with 18–25 bases made up of unmethylated CG motifs (cytosine phosphate guanidine), which are recognised by endosomal Toll Like Receptor 9 (TLR9). The CpG ODN are the agonists of TLR9 which are predominantly present in B cells, macrophages, monocytes and dendritic cells [10]. Human and other vertebrates detect the unmethylated CpG motif or synthetic ODN as a sign of danger signals [11,12] and activates innate and adaptive immune response in the host [13]. The CpG acts on the cells that express TLR9 and initiate maturation, differentiation and proliferation of natural killer cells (NK cells), T cells and macrophages, leading to secretion IL-12, TNF-α and type-I IFN (IFN-α & IFN-β) [14]. Several studies involving CpG as a vaccine adjuvant have produced better humoral and cell mediated immune responses [15] and CpG has been approved as an adjuvant in human vaccines like Hepatitis-B (HEPLISAV-B) [16] and Influenza vaccines.

Considering the merits of CpG as an adjuvant and the limitations of the currently available non-adjuvanted KFD vaccine, we formulated CpG adjuvanted Kyasanur Forest Disease vaccine and conducted its immunological efficacy in mice model.

## Materials and methods

### Confirmation of Kyasanur Forest Disease (KFD) seed virus

Kyasanur Forest Disease seed virus (P9605) used in this study for the production of KFD vaccine was procured from ICMR- National Institute of Virology (NIV) Pune. The virus was provided in mouse brain suspension. The seed virus was confirmed by TaqMan probe-based one-step real-time reverse transcription PCR. The KFD viral RNA was extracted from infected mouse brain suspension as per QIAamp® RNA Blood Mini kit. The extracted RNA was subjected for one-step real-time reverse transcription PCR. The primers, probe, master mix and the cycling conditions described by Mourya and co-workers [17] were used in this study. The primers used in this study included Forward primer 5’ TGGAAGCCTGGCTGAAAGAG 3’ Reverse primer 5’ TCATCCCCACTGACCAGCAT 3’ and the TaqMan probe 5’ ATGGAGAGGAGCGCCTGACCCG 3’. The PCR cycling conditions included, reverse transcription at 50°C for 30 min and Taq inhibitor inactivation at 95°C for 10 min followed by 40 cycles of 95°C for 15 Sec and 60°C for 1 min. The results were recorded at the end of 40 cycles of amplification. The PCR reactions were carried out in a QIAGEN QIAquant-96 real-time PCR system at the KFD vaccine laboratory of Institute of Animal Health and Veterinary Biologicals, Bangalore, India.

### Production of formalin inactivated KFD vaccine

The KFD vaccine used in this study was prepared by growing the KFD seed virus P9605 in chick embryo fibroblast primary culture. The KFD seed virus used for vaccine production was mouse brain suspension (passage 4) containing KFD virus of titre of MLD_50_ 10 ^7^/ 0.2 ml.

### Vaccine production in Chick embryo fibroblast monolayer culture

Primary chick embryo fibroblast (CEF) culture was prepared from 9-day-old chick embryos as per standard protocols [1,6,8]. The CEF monolayers in each Roux flask were infected with 0.01 multiplicity of infection (MoI) of KFDV by adsorption method at 37 ºC for 1 hour. The Roux flasks were incubated at 37 ± 1 ºC for 48 hours. First virus harvest (H1) of the cell culture supernatant was done at 48 hours post-infection and subsequent four harvests (H2, H3, H4 and H5) were done at every 24 hours interval after the first harvest.

Following procedures were performed at each harvest.

a) Aliquots of virus harvests were subjected for sterility check as per standard protocols [6,8].
b) Aliquots of each harvest were collected and stored at −80 ºC for determination of KFD virus titre in pre-inactivated cell culture supernatant.
c) The harvested flasks were replenished with fresh maintenance medium and incubated at 37 ± 1ºC
d) Finally, the collected virus harvests were inactivated with formalin as described below;

### Inactivation of the KFD virus

Based on the quantity of the cell culture supernatant/virus harvests collected, formalin was added such that the final concentration of formalin in the vaccine is 0.1% [1,6,8]. The containers with the cell culture virus harvest with formalin were kept in a shaker incubator at 4°C for 14 days. The formalin inactivated KFD vaccine was clarified by passing through a microfiltration unit comprising of different filter cassettes of 0.8 µ, 0.6 µ and lastly through 0.2 µ filter.

### Determination of the vaccine titre

The KFD seed virus used for vaccine production in this study was non-cytopathic in cell-culture indicator system and titre had to be determined by *in-vivo* tests only. Hence, the titre of the vaccine produced was determined in mice as per standard protocols [1,6,8]. For this, the aliquots of pre-inactivated virus harvests collected during each virus harvest were pooled and subjected for determination of virus titre (Mouse lethal dose-MLD_50_) by mouse inoculation test [1]. The titre of the vaccine was adjusted to have a log protective index of ≥5.4 per dose of vaccine used [6,8].

### Mice strain used in the study

The mice used in this entire study belonged to Swiss albino (*Mus musculus*) strain. During all mice experiments, mice were observed by qualified veterinarians at every 12-hour intervals for their health, behavior and disease symptoms (if any).

#### Determining the effective dose of CpG for adjuvantation with KFD vaccine by mouse protection test.

Mouse protection test was used to determine the effective dose of the CpG for adjuvantation with KFD vaccine in mice as per the previously described procedures [1,4,6,18]. Briefly, forty-two mice of 3–4 weeks old were divided into seven groups. The first group (n = 6) was inoculated with 0.5 ml of KFD vaccine containing 2 ug of CpG; the second group (n = 6) was immunised with 0.5 ml of KFD vaccine containing 4 ug of CpG; the third group (n = 6) was immunised with 0.5 ml of KFD vaccine containing 6 ug of CpG; the fourth group (n = 6) was immunised with 0.5 ml of KFD vaccine containing 8 ug of CpG; the fifth group (n = 6) was immunised with 0.5 ml of KFD vaccine containing 10 ug of CpG; the sixth group (n = 6) was immunised with 0.5 ml of non-adjuvanted KFD vaccine. The immunisations were done on 0 day and 21^st^ day, by subcutaneous route. The seventh group (n = 6) was inoculated with 0.5 ml of PBS and remained as unvaccinated control. On 28^th^ day after first dose of vaccination mice from each group were euthanized using Isoflurane and heart blood was collected from mice. Serum was separated by centrifugation at 2000 rpm for 5 minutes, separated serum from each group was pooled and filtered through 0.2 μm syringe filter. Filtered serum was heat inactivated at 56°C for 30 min and subjected for mouse protection test, as described previously [1,4,6,18].

During the mouse protection test, KFD symptoms of hind quarter paralysis and inability to move were taken as endpoint symptoms for computing the survival graphs. Mice showing symptoms of hind quarter paralysis and inability to move were humanely euthanized.

### Determining the safe dose of CpG adjuvanted KFD vaccine

The CpG adjuvant (CpG ODN 1585 VacciGrade, referred as CpG in this entire paper) used in this study was procured from *M/s Invivogen*, USA. The KFD vaccine was adjuvanted with three different concentrations of CpG adjuvant viz., 2 μg, 5 μg and 10 μg per dose of vaccine. The adjuvanted vaccine from each concentration was inoculated into 3–4-week-old mice (n = 6) by subcutaneous (s/c) route. Each mouse in three different groups received 1.0 ml of CpG adjuvanted vaccine (double the dose of the vaccine and double the dose of CpG adjuvant given in mice). The inoculated mice were observed at every 12-hour intervals for 21 days for symptoms of illness/inflammation/abnormalities (Table 2). The vaccine with highest concentration of CpG adjuvant which did not produce any symptoms of illness was selected for further vaccination studies.

**Table 1:** Survival percentage of mice in KFD vaccine adjuvanted with different concentrations of CpG adjuvant (2 ug, 4 ug., 6 ug, 8 ug and 10 ug) per dose of vaccine, non-adjuvanted KFD vaccine and control group in the Mouse Protection Test for determining the effective dose of CpG adjuvant to be used in the KFD vaccine in mice.

Serum Dilutions	KFD Vaccine adjuvanted with CpG adjuvant (per dose) (Survival %)					Non-Adjuvanted KFD Vaccine Group (Survival %) (n = 6)	Unvaccinated Control Group (Survival %) (n = 6)
	2 ug of CpG (n = 6)	4 ug CpG (n = 6)	6 ug CpG (n = 6)	8 ug CpG (n = 6)	10 ug CpG (n = 6)		
**1:2**	6/6 = 100%	6/6 = 100%	6/6 = 100%	6/6 = 100%	6/6 = 100%	6/6 = 100%	0/6 = 0%
**1:4**	2/6 = 33.33%	2/6 = 33.33%	4/6 = 66.66%	6/6 = 100%	6/6 = 100%	2/6 = 33.33%	0/6 = 0%
**1:8**	0/6 = 0%	0/6 = 0%	2/6 = 33.33%	4/6 = 66.66%	4/6 = 66.66%	0/6 = 0%	0/6 = 0%
**1:16**	0/6 = 0%	0/6 = 0%	0/6 = 0%	2/6 = 33.33%	2/6 = 33.33%	0/6 = 0%	0/6 = 0%
**1:32**	0/6 = 0%	0/6 = 0%	0/6 = 0%	0/6 = 0%	0/6 = 0%	0/6 = 0%	0/6 = 0%

**Table 2:** Safety test for 2 μg, 5 μg and 10 μg CpG adjuvanted KFD vaccine in mice.

Days	CpG adjuvanted KFD vaccine								
	2 μg of CpG adjuvant			5 μg of CpG adjuvant			10 μg of CpG adjuvant		
	1 ml s/c (n = 6)			1 ml s/c (n = 6)			1 ml s/c (n = 6)		
	H	S	D	H	S	D	H	S	D
Day 1	6	–	–	6	–	–	6	–	–
Day 2	6	–	–	6	–	–	6	–	–
Day 3	6	–	–	6	–	–	6	–	–
Day 4	6	–	–	6	–	–	6	–	–
Day 5	6	–	–	6	–	–	6	–	–
Day 6	6	–	–	6	–	–	6	–	–
Day 7	6	**–**	–	6	–	–	6	–	–
Day 8	6	–	–	6	–	–	6	–	–
Day 9	6	–	–	6	–	–	6	–	–
Day 10	6	–	–	6	–	–	6	–	–
Day 11	6	–	–	6	–	–	6	–	–
Day 12	6	–	–	6	–	–	6	–	–
Day 13	6	–	–	6	–	–	6	–	–
Day 14	6	–	–	6	–	–	6	–	–
Day 15	6	–	–	6	–	–	6	–	–
Day 16	6	–	–	6	–	–	6	–	–
Day 17	6	–	–	6	–	–	6	–	–
Day 18	6	–	–	6	–	–	6	–	–
Day 19	6	–	–	6	–	–	6	–	–
Day 20	6	–	–	6	–	–	6	–	–
Day 21	6	–	–	6	–	–	6	–	–

**Note:** H – Healthy; S – Symptoms; D – Death.

### Evaluation of humoral immune response by mouse protection test

Mouse protection test was used to assess the humoral immune response (HIR) in vaccinated mice, as per the previously described procedures [6,18]. Briefly this involved,

a) Vaccination of mice and collection of serum samples and
b) Virus neutralisation and protection test in mice
a) **Vaccination of mice and collection of serum samples**

Mice (3–4-week-old) were immunised with CpG adjuvanted KFD vaccine and non-adjuvanted KFD vaccine. One group of mice (n = 6) was immunised with 0.5 ml KFD vaccine containing 10 ug of CpG, second group (n = 6) was immunised with 0.5 ml of non-adjuvanted KFD vaccine on 0 day and 21^st^ day and third group were inoculated with 0.5 ml of PBS by subcutaneous route. Mice from each group were euthanized using Isoflurane on 28^th^ day and heart blood was collected from mice. Serum was separated by centrifugation at 2000 rpm for 5 minutes, separated serum from each group was pooled and filtered through 0.2 μm syringe filter. Filtered serum was heat inactivated at 56°C for 30 min and subjected for mouse protection test.

b) **Virus neutralisation and protection test in mice**

Two-fold dilution of serum from each group was prepared (0.75 ml of mice serum from each group + 0.75 ml of MEM plain media) from 1:2–1:32. To each serially diluted serum, 0.75 ml of 1MLD_50_ challenge virus was added and incubated at 37°C for one hour. After incubation, 0.2 ml of serum-virus mixture from each dilution were injected to suckling mice by intraperitoneal (i/p) route and mice were observed for 14 days for symptoms of KFD and/or death. The KFD symptoms of hind quarter paralysis and inability to move were taken as endpoint symptoms for computing the survival graphs. Mice showing symptoms of hind quarter paralysis and inability to move were humanely euthanized.

### Assessment of Cell Mediated Immune (CMI) response/ Cytokine response

Mice (3–4-week-old) were immunised with CpG adjuvanted KFD vaccine and non-adjuvanted KFD vaccine and CMI response/ cytokine response was assessed by estimation of Interferon-Gamma (IFN- ϒ), Tumour Necrosis Factor-Alpha (TNF-α) and IL-12 in the plasma of immunised mice.

One group of mice (n = 6) was immunised with 0.5 ml KFD vaccine containing 10 ug of CpG, second group (n = 6) was immunised with 0.5 ml of non-adjuvanted KFD vaccine on 0 day and 21^st^ day and third group were inoculated with 0.5 ml of PBS by subcutaneous route. Mice from each group were euthanized using Isoflurane on 28^th^ day and heart blood was collected from mice. Twenty-four well plates were used to separate plasma from the collected blood samples. The blood collected from each mouse was placed in three wells in the 24 well plate, wherein the blood in one well was stimulated with 1MLD_50_ KFD virus, the second well was stimulated with Concavalin-A (at the rate of 1.0 mg/ml) which served as positive control and one well was left unstimulated which served as negative control. The plate was incubated in CO_2_ incubator with 5% CO2 for 24 hrs. The plasma was subjected for estimation of IFN-ϒ, TNF-α and IL-12 production by enzyme linked immune sorbent assay (ELISA) using ELISA kits (Mabtech, USA) procured from M/s Aadinath Veterinary Products Private Limited, India.

### Potency test procedure

Potency test was carried out in ninety mice of 3–4-week-old which were divided into three groups of 30 mice each. One group of mice was immunised with 0.5 ml of CpG adjuvanted Kyasanur forest disease vaccine by subcutaneous route (s/c) on 0 day and 7^th^ day. Second group of mice was immunised with 0.5 ml of non-adjuvanted KFD vaccine by subcutaneous (s/c) route on 0 day and 7^th^ day. Thirty mice of the same age were left as unvaccinated control. On 14th day after first dose of vaccination, the KFD challenge virus was titrated in adjuvanted, non-adjuvanted and control mice by inoculating ten-fold serially diluted virus in a group of six mice, each mouse receiving 0.2 ml of KFD virus by i/p route. The dilutions of the KFD challenge virus inoculated in adjuvanted and non-adjuvanted vaccine group were 10^−1^ to 10^−5^ whereas, control group were inoculated with 10^−5^ to 10^− 9^ dilutions (Table 2) (we did not inoculate 10^−1^ to 10^−5^ dilutions in control mice as all mice will die with these higher dilutions KFD virus). The log MLD_50_ titres of virus in CpG adjuvanted, non-adjuvanted and control group was calculated by using Reed and Muench formula [19]. Difference between the logarithmic titres of control and immunized mice was deduced as log protective index, as per the protocol of Dandawate [6]. The KFD symptoms of hind quarter paralysis and inability to move were taken as endpoint symptoms for computing the survival graphs. Mice showing symptoms of hind quarter paralysis and inability to move were humanely euthanized [4].

### Statistical analyses

Statistical analyses were performed using GraphPad software (La Jolla, CA). All comparisons were made using an one-way ANOVA test with Tukey corrected multiple comparisons or Students t test where P<0.05 = *, P<0.005 = **, P<0.0005 = *** were considered significantly different among groups. Survival Graphs were plotted using Kaplan-Meier survival curves and statical significance in survival graphs were plotted by Log-rank test. Data are presented as mean ± SEM for biological replicates. Viral titres were log transformed prior to analysis. No data or outliers were excluded from analysis.

## Results

### Confirmation of the Kyasanur Forest Disease (KFD) Seed Virus

The KFD seed virus (P9605) used for the production of the KFD vaccine was confirmed by Real time RT-PCR. The Real time RT-PCR yielded amplification of KFD virus with threshold cycle (CT) values of 14 confirming the presence of KFDV in mice brain suspension.

### Determining the effective dose of CpG for adjuvantation with KFD vaccine

The mouse protection test was performed to determine the effective dose of CpG for adjuvantation and showed that CpG when used at the rate of 8 ug or 10 ug per dose of KFD vaccine had given the best protection levels in mice (Table 1).

### Safety test for CpG adjuvanted KFD vaccine

The KFD vaccine was adjuvanted with three different concentrations of CpG viz., 2 μg, 5 μg and 10 μg per dose of vaccine. The adjuvanted vaccine from each concentration was inoculated into 3–4 week old mice (n = 6) by subcutaneous (s/c) route. Each mouse in three different groups received 1.0 ml of CpG adjuvanted vaccine. Over the course of 21 days, the mice were closely monitored for illness, symptoms or any adverse side effects. Each mouse in three different CpG adjuvanted vaccine groups were healthy and did not exhibit any adverse reactions during the study period. All the three different concentrations (2 μg, 5 μg and 10 μg) of CpG adjuvant had passed the safety test (Table 2). The highest concentration of the CpG adjuvant (10 μg) that passed the safety test was used for further vaccine evaluation.

### Evaluation of humoral immune response by mouse protection test

**Fig 1:**
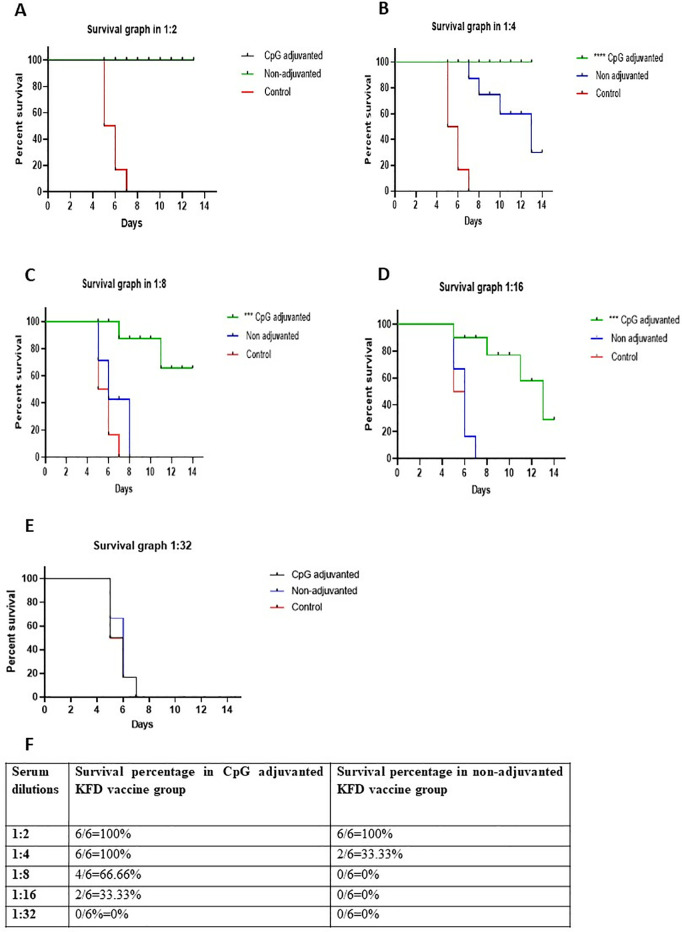
Graphical representation of the survival percentage of mice in CpG adjuvanted KFD vaccine, non-adjuvanted KFD vaccine and control group in the Mouse Protection Test for evaluation of Humoral Immune Response. A: Survival graph in 1:2 dilution of serum. B: Survival graph in 1:4 dilution of serum. C: Survival graph in 1:8 dilution of serum. D Survival graph in 1:16 dilution of serum. E: Survival graph in 1:32 dilution of serum. F: Summary of the survival percentage of mice in mouse protection test to evaluate the humoral immune response in CpG adjuvanted and non-adjuvanted KFD vaccine for mice serum. Note: *, **, ***, **** indicate significance at *P* < 0.1, 0.01, 0.001 and 0.0001, respectively.

The humoral immune response was evaluated by mouse protection test. There was 100% protection in both CpG adjuvanted and non-adjuvanted KFD vaccine received groups in 1:2 diluted serum (Fig 1A). In 1:4 diluted serum 100% and 33.33% protection was observed in CpG adjuvanted KFD vaccine group and non-adjuvanted KFD vaccine group, respectively (Fig 1B). In 1:8 dilution of serum 66.66% of mice were protected in CpG adjuvanted KFD vaccine group and no mice survived in non-adjuvanted KFD vaccine group (Fig 1C). In 1:16 serum dilution 33.3% of mice survived in CpG adjuvanted KFD vaccine group and no mice survived in non-adjuvanted KFD vaccine group (Fig 1D). In 1:32 dilutions no mice survived in both CpG adjuvanted and non-adjuvanted KFD vaccine groups (Fig 1E). The survival percentages conferred by different serum dilutions in CpG adjuvanted and non-adjuvanted groups are shown in Fig 1F.

The statistical analysis revealed that CpG adjuvanted KFD vaccine showed significantly better protection in mice compared to non-adjuvanted KFD vaccine (P < 0.0001) in 1:4, 1:8 and 1:16 dilutions of serum. No significant protection was observed in 1:32 dilution of serum between CpG adjuvanted KFD vaccine and non-adjuvanted KFD vaccine.

### Assessment of Cell Mediated Immune (CMI) response/ Cytokine response

**Fig 2:**
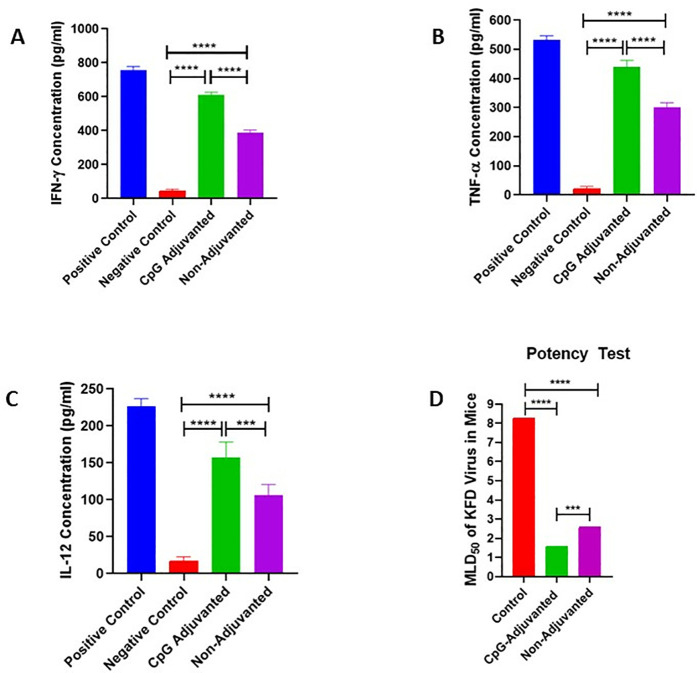
A: Concentration of IFN-ϒ in mice groups vaccinated with CpG adjuvanted KFD vaccine and non-adjuvanted KFD vaccine and the control groups. B: Concentration of TNF-α in mice groups vaccinated with CpG adjuvanted KFD vaccine and non-adjuvanted KFD vaccine and the control groups. **C:** Concentration of IL-12 in mice groups vaccinated with CpG adjuvanted KFD vaccine and non-adjuvanted KFD vaccine and the control groups. **D**: The MLD_50_ of KFD challenge virus in CpG adjuvanted KFD vaccine group, non-adjuvated. KFD vaccine group and control group in potency test in mice. Note: *, **, ***, **** indicate significance at *P* < 0.1, 0.01, 0.001 and 0.0001, respectively.

The production of IFN-ϒ, TNF-α and IL-12 in mice showed that the plasma samples from mice immunized with CpG adjuvanted KFD vaccine had significantly (p < 0.0001) higher levels of IFN-ϒ (Fig 2A), TNF-α (Fig 2B) and IL-12 (Fig 2C) compared to the mice immunized with non-adjuvanted KFD vaccine, demonstrating the ability of CpG to enhance CMI response and cytokines when used as an adjuvant with KFD vaccine.

### Assessment of protective efficacy of CpG adjuvanted vaccine by potency test

Potency test was performed in three groups of mice, CpG adjuvanted KFD vaccine group, non-adjuvanted KFD vaccine group and control group (Table 3). It was observed that

**Table 3:** Potency test for CpG adjuvanted and non-adjuvanted KFD vaccine.

Potency test for CpG adjuvanted and non-adjuvanted KFD vaccine in mice																	
Days	CpG adjuvanted KFD vaccine group (n = 30)					Non-adjuvanted KFD vaccine group (n = 30)					Control group (n = 30)					Media only	Uninoculated control
Day 0	1^st^ dose of vaccine					1^st^ dose of vaccine											
Day 7	2^nd^ dose of vaccine					2^nd^ dose of vaccine											
	10^−1^	10^−2^	10^−3^	10^−4^	10^−5^	10^−1^	10^−2^	10^−3^	10^−4^	10^−5^	10^−5^	10^−6^	10^−7^	10^−8^	10^−9^	(n = 6)	(n = 6)
	(n = 6)	(n = 6)	(n = 6)	(n = 6)	(n = 6)	(n = 6)	(n = 6)	(n = 6)	(n = 6)	(n = 6)	(n = 6)	(n = 6)	(n = 6)	(n = 6)	(n = 6)		
Day 14	6	6	6	6	6	6	6	6	6	6	6	6	6	6	6	6	6
Day 15	6	6	6	6	6	6	6	5	6	6	5	5	6	6	6	6	6
Day 16	6	6	6	6	6	5	6	5	6	6	5	5	5	6	6	6	6
Day 17	4	6	6	6	6	5	6	5	6	6	4	5	5	6	6	6	6
Day 18	4	6	6	6	6	5	6	5	6	6	4	4	4	5	6	6	6
Day 19	4	6	6	6	6	5	6	5	6	6	4	3	4	5	5	6	6
Day 20	3	6	6	6	6	4	6	5	6	6	3	3	3	5	5	6	6
Day 21	3	6	6	6	6	4	6	5	6	6	3	3	3	5	5	6	6
Day 22	3	6	6	6	6	2	6	5	6	6	2	3	3	5	5	6	6
Day 23	2	6	6	6	6	2	5	4	6	6	2	3	2	4	5	6	6
Day 24	2	6	6	6	6	1	3	4	6	6	2	3	1	4	5	6	6
Day 25	1	6	6	6	6	0	3	4	6	6	1	2	1	3	4	6	6
Day 26	0	5	6	6	6	0	3	4	6	6	0	1	1	2	4	6	6
Day 27	0	5	6	6	6	0	3	4	6	6	0	1	1	2	4	6	6
Day 28	0	5	6	6	6	0	3	4	5	6	0	1	1	2	4	6	6

the MLD_50_ of KFD challenge virus in mice given with 10 μg of CpG adjuvanted KFD vaccine was 10^-1.602^/0.2 ml (Tables 3 and 4) (Fig 2D). The MLD_50_ of KFD challenge virus in mice given with non-adjuvanted KFD vaccine was 10^-2.459^/0.2 ml (Tables 3 and 5) (Fig 2D). The MLD_50_ of KFD challenge virus in unvaccinated mice was 10^-8.25^/0.2 ml (Tables 3 and 6) (Fig 2D).

**Table 4:** Virus titre (MLD_50_) in CpG adjuvanted KFD vaccine group.

CpG adjuvanted KFD vaccine group							
Virus Dilution	Death Ratio	Number of Death	Number of Live	Cumulative		Death (Accumulate)	% Mortality
				(D)	(L)		
10^−1^	6/6	6	0	7	0	7/7	100%
10^−2^	1/6	1	5	1	5	1/6	17%
10^−3^	0/6	0	6	0	11	0/11	0%
10^−4^	0/6	0	6	0	17	0/17	0%
10^−5^	0/6	0	6	0	23	0/23	0%

**Table 5:** Virus titre (MLD_50_) in Non-adjuvanted KFD vaccine group.

Non-adjuvanted KFD vaccine group							
Virus Dilution	Death Ratio	Number of Death	Number of Live	Cumulative		Death (Accumulate)	% Mortality
				(D)	(L)		
10^−1^	6/6	6	0	12	0	12/12	100%
10^−2^	3/6	3	3	6	3	6/9	67%
10^−3^	2/6	2	4	3	7	3/10	30%
10^−4^	1/6	1	5	1	12	1/13	7%
10^−5^	0/6	0	6	0	18	0/18	0%

**Table 6:** Virus titre (MLD_50_) in Control group.

Control group							
Virus Dilution	Death Ratio	Number of Death	Number of Live	Cumulative		Death (Accumulate)	% Mortality
				(D)	(L)		
10^−5^	6/6	6	0	22	0	22/22	100%
10^−6^	5/6	5	1	16	1	16/17	94%
10^−7^	5/6	5	1	11	2	11/13	84%
10^−8^	4/6	4	2	6	4	6/10	60%
10^−9^	2/6	2	4	2	8	2/10	20%

The logarithmic titres of the virus in CpG adjuvanted, non-adjuvanted and control group of mice were 10^-1.602^, 10^-2.459^ and 10^-8.25^ respectively (Fig 2D). The logarithmic titres of the KFD challenge virus were determined by Reed and Muench method [19] and the results showed that mice immunised with CpG adjuvanted KFD vaccine had superior ability to resist KFD challenge virus compared to the mice immunised with non-adjuvanted KFD vaccine.

The Log Protective index was calculated as per NIV guidelines and it is defined as the difference in logarithmic titre MLD_50_ in control mice and the vaccinated mice [1].

### Log protective index of CpG adjuvanted KFD vaccine


Log\Protective\index = Difference\ in\ logarithmic\ titers\ of\ control\ and\ immunized\ mice

$$=\text{ Log }{\text{MLD}}_{50}\text{ control }–\text{ Log }{\text{MLD}}_{50}\text{ CpG\textbackslash{} adjuvanted\textbackslash{} KFD\textbackslash{} vaccine}$$

$$=\;10^{-8.25}\;-\;10^{-1.602}\;=\;10^{-6.648}$$

### Log protective index of non-adjuvanted KFD vaccine


$$\text{Log\textbackslash{}Protective\textbackslash{}index}=\text{ Log }{\text{MLD}}_{50}\text{ control }–\text{ Log }{\text{MLD}}_{50}\text{ non - adjuvanted\textbackslash{} KFD\textbackslash{} vaccine}$$

$$=\;10^{-8.25}\;-\;10^{-2.459}\;=\;10^{-5.791}$$

In this study the log protective index of CpG adjuvanted KFD vaccine and non-adjuvanted KFD vaccine was 10^-6.648^ and 10^-5.791^ respectively. The results showed that the protective index (PI = 6.648) in CpG adjuvanted KFD vaccine group was 0.8 log more than the protective index (PI = 5.8) in group given with non-adjuvanted KFD vaccine, indicating that the adjuvanted KFD vaccine is more potent compared to the non-adjuvanted KFD vaccine.

**Fig 3:**
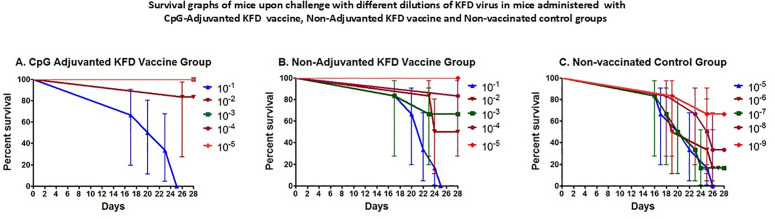
Potency test was performed in three groups of mice, CpG adjuvanted KFD vaccine group, non adjuvanted KFD vaccine group and control group. Thirty mice in group-I were inoculated with 0.5 ml of CpG adjuvanted KFD vaccine by subcutaneous (s/c) route on 0 day and 7^th^ day, non-adjuvanted KFD vaccine was inoculated to 30 mice in second group by subcutaneous (s/c) route on 0 day and 7^th^ day. Thirty mice of same age were left as unvaccinated control. On 14^th^ day after 1^st^ dose of vaccination, the challenge KFD virus was titrated in adjuvanted, non-adjuvanted and control mice by inoculating 10-fold serially diluted virus in a group of 6 mice, each mice receiving 0.2 ml of KFD virus by i/p route. The dilutions of the challenge KFD virus inoculated in adjuvanted and non-adjuvanted vaccine groups are 10^−1^ to 10^−5^ whereas the control group was inoculated with 10^−5^ to 10^−9^ dilutions. The results in the survival graph show that in group receiving CpG adjuvanted KFD vaccine (A), there was 0% of mice survived when challenged with 10^−1^ of KFD virus, 90% mice survived upon challenge with 10^−2^ KFD challenge virus and 100% mice survived in mice given with 10^−3^, 10^−4^ and 10^−5^ dilutions of the KFD challenge virus, respectively. In the mice group receiving non-adjuvanted KFD vaccine (B), the survival parentages were 0%, 50%, 70%,90% and 100% in mice receiving 10^−1^, 10^−2^ 10^−3^, 10^−4^ and 10^−5^ dilutions of the KFD challenge virus, respectively. In non-vaccinated control group the survival percentage of mice were 0%, 20%, 20%, 33.33% and 66.66% upon challenge with 10^−5^, 10^−6^,10^−7^, 10^−8^ and 10^−9^ dilutions of the KFD challenge virus, respectively.


Survival graphs of mice upon challenge with different dilutions of KFD challenge virus depicted better survival percentages in mice immunised with CpG-adjuvanted KFD vaccine compared to mice immunised with non-adjuvanted KFD vaccine (Fig 3).

$$\text{PD}=\;\frac{\text{Infectivity\textbackslash{} above }50\%\;-50}{\text{Infectivity\textbackslash{} above }50\%\;-\text{ Infectivity\textbackslash{} below }50\%}=\;\frac{100-50}{100-17}\;=\;\frac{50}{83}=\;0.602$$

$$\;{\text{MLD}}_{50}={\;10}^{-1.602}/0.2\text{ ml}$$

$$\text{PD}=\;\frac{\text{Infectivity\textbackslash{} above }50\%\;-50}{\text{Infectivity\textbackslash{} above }50\%\;-\text{ Infectivity\textbackslash{} below }50\%}=\;\frac{67-50}{67-30}=\;\frac{17}{37}=\;0.459\;$$

$${\text{MLD}}_{50}=10^{-2.459}/0.2\text{ ml}$$

$$\text{PD}=\;\frac{\text{Infectivity\textbackslash{} above }50\%\;-50}{\text{Infectivity\textbackslash{} above }50\%\;-\text{ Infectivity\textbackslash{} below }50\%}=\;\frac{60-50}{60-20}=\;\frac{10}{40}=\;0.25$$

$${\text{MLD}}_{50}=\;10^{-8.25}/0.2\text{ ml}$$

## Discussion

In 1957, Kyasanur forest of Shimoga district, Karnataka state in India witnessed unusually high mortality in monkeys with concurrent outbreaks in humans. National institute of Virology (formerly known as Virus Research Centre, Pune) isolated the virus and named the disease as Kyasanur Forest Disease (KFD). With many scientific challenges, Indian scientists developed a formalin inactivated tissue culture KFD vaccine in 1960s [5] using the KFD seed virus P9605. In 1970–71 the efficacy of KFD vaccine had demonstrated antibody response in 59% of the vaccinated individuals and the vaccine had protected against the disease occurrence and severity compared to the unvaccinated individuals [20]. The epidemiological survey by Kasabi and co-workers [7] who evaluated the KFD vaccine efficacy between 2005–2010 revealed 62.4% effective in individuals who received two doses and 82.9% effective in those who received two doses followed by a booster dose when compared to unvaccinated individuals.

The KFD vaccine produced at IAH &VB, Bengaluru since 2000 has helped in significantly reducing the morbidity (1832 cases and 38 deaths) from 2000–2005–2016–2020 (787 cases and 6 deaths) [1,4]. Taking together the recent molecular epidemiological data, the number of KFD cases and deaths have drastically reduced from 1980s to 2020 with rigorous vaccination campaigns in endemic areas suggesting that the seed virus used for vaccine production is still relevant and effective at the ground level. Considering these factors KFD seed virus P9605 was used in this study for development of a new adjuvanted vaccine.

Vaccination remains the single most effective method of preventing infectious diseases. When a host organism encounters a viral or bacterial pathogen, recognition of the pathogen is achieved primarily through early sentinels called Pattern Recognition Receptor (PRR) molecules that bind with Pathogen Associated Molecular Patterns (PAMPS) [21]. Today, one of the best studied PRR is Toll Like Receptor (TLR) family, which are transmembrane signalling molecules that play a key role in the initiation of innate immune responses and also influence antigen specific adaptive immune responses. The most prominent and well characterised TLR within this subset is TLR9 which shows restricted and species-specific pattern of cellular distribution. A CpG dinucleotide is an absolute requirement for TLR9 activation. Synthetically manufactured oligodeoxynucleotide (ODN) sequences engineered with one or more CpG motif can also activate TLR9 expression. It is now well established that humans and other vertebrates may detect unmethylated CpG (CpG motifs) or synthetic ODN as a sign of danger and mimic a natural infection activating a coordinated set of immune responses that include innate immunity, humoral immunity and cellular immunity.

Three concentrations of CpG *viz*., 2 μg, 5 μg and 10 μg per dose of vaccine in mice were evaluated for safety parameters. All the three concentrations when used by subcutaneous route with double the normal dose of vaccine in mice did not cause any adverse reactions or symptoms of disease or discomfort in mice under the safety test. The results were in accordance with the previous works [22,23] who have studied CpG as an adjuvant in mice and non-human primates without any microscopic and macroscopic evidence of tissue damage or inflammation or adverse effects.

The humoral immune response (HIR) of the CpG adjuvanted vaccine was assessed by mouse protection test. The mice immunised with CpG adjuvanted KFD vaccine had significantly better HIR with higher survival percentages compared to the mice immunised with non-adjuvanted KFD vaccine. The comparatively better HIR in CpG adjuvanted group could be attributed to preferential expression of TLR9 in B lymphocytes in mice and possibly, CpG has directly activated the TLR9 pathway of immune activation of B cells in those mice vaccinated with CpG adjuvanted KFD vaccine [24] and CpG DNA directly activating B cell proliferation to increase the secretion of IL-6 and immunoglobulins to increase the cell surface expression of costimulatory molecules [25]. The findings were in accordance with previous works who have demonstrated significantly higher and faster antibody response when CpG was used as an adjuvant in Hepatitis B vaccine in mice and humans [26–28], SARS CoV vaccine in mice [29], anthrax vaccine [30], influenza virus vaccine [31], and tetanus toxoid [32].

The cell mediated immune response was evaluated in mice that were vaccinated twice with or without adjuvant and were assessed for IFN-γ, TNF-α, IL-12, production using ELISA; since IFN- γ is one of the most important functional attributes of a successful CMI response mediated by activated T-lymphocytes [33], IL-12, TNF-α are important proinflammatory cytokines that mediate both innate and cellular immune responses in host [14,16,24]. The ELISA results showed that CpG adjuvantation of KFD vaccine had enhanced IFN-ϒ and TNF-α production in mice. This could be attributed to CpG DNA mimicking a natural infection characterized by maintenance of cellular composition instigating an intense CMI response with Th1 polarized response [11,13,14]. The findings in this study were in accordance with the enhanced CMI response when CpG is used as an adjuvant in vaccines for Hepatitis-B (27), *Staphylococcus aureus* [34], SARS COVID-19 [35], Influenza [33,36], *Listeria monocytogenes* vaccine [37].

The IFN-ϒ production by T lymphocytes can be induced after recognition of specific antigen by these cells. However, production of IFN-ϒ by NK cells is not antigen specific and is primarily driven by IL-12 produced by macrophages/dendritic cells [24]. The unmethylated CpG motifs activates macrophages and induces substantial production of IL-12 which is responsible for driving NK cells to produce profound quantities of IFN-ϒ helping in clearing the intra-cellular pathogen and enhancing the protection [24,25].

The potency and efficacy of the CpG adjuvanted KFD vaccine was assessed by challenge studies. The KFD challenge virus was titrated in vaccinated and control mice. The protective index (PI = 6.648) in CpG adjuvanted group was 0.8 log more than the protective index (PI = 5.8) in group given with non-adjuvanted KFD vaccine. This could be because the CpG DNA activates strong innate and adaptive immune system due to preferential localization of TLR9 receptors in monocytes, macrophages and dendritic cells which are the contributors of innate immune response and the B cells which are the main cells of humoral type of adaptive immune system (28). Further, CpG activates macrophages, monocytes and dendritic cells to produce type-I interferons and particularly activates plasmacytoid dendritic cells (pDC’s) to produce large quantities of type-I interferons (IFN-α and IFN-β). It is important to note that pDC’s are the highest producers of type-I IFN in the body [38]. The major way by which the innate immune system blocks viral infections is to induce type-I interferons that inhibits viral replication [39]. The better protective index achieved in CpG vaccinated group is attributed to overall enhancement of innate immune system through production of IL-12 and type-I IFN by dendritic cells and enhancing the NK cell activity leading to increased IFN-γ production and lytic activities combined with a significantly improved adaptive HIR as described previously.

The study demonstrated that CpG induces increased production inflammatory cytokines like IL-12, TNF-α; enhances antiviral response by increasing production of IFN-ϒ and enable better humoral immune response when used as an adjuvant in KFD vaccine in mice eventually leading to better protection against KFD virus. The results in this study encourages to further test this CpG adjuvanted KFD vaccine in monkeys and in humans before a new generation vaccine like VSV vectored recombinant vaccine for KFD which has shown safe and strong adaptive immune responses against KFDV infection in a rodent and nonhuman primate model [40] is available for human use.
